# The effectiveness of healthy lifestyle interventions on weight gain in overweight pregnant women: A cluster‐randomized controlled trial

**DOI:** 10.1002/nop2.577

**Published:** 2020-08-02

**Authors:** Sepideh Hajian, Armin Aslani, Parvin Sarbakhsh, Azita Fathnezhad‐Kazemi

**Affiliations:** ^1^ Department of Midwifery & Reproductive Health Faculty of Nursing & Midwifery Shahid Beheshti University of Medical Sciences Tehran Iran; ^2^ Medical student, Student Research Committee, Tabriz Branch Islamic Azad University Tabriz Iran; ^3^ Department of Statistics and Epidemiology School of Public Health Tabriz University of Medical Sciences Tabriz Iran; ^4^ Department of Midwifery Faculty of Nursing and Midwifery, Tabriz Branch Islamic Azad University Tabriz Iran

**Keywords:** consultation, lifestyle, nutrition, overweight, physical activity, pregnancy

## Abstract

**Aim:**

Interventions based on adopting a healthy lifestyle have been less successful. The aim of this study was to investigate the effectiveness of healthy lifestyle interventions on weight gain in overweight pregnant women.

**Design:**

A cluster randomized controlled trial.

**Methods:**

Health centres were selected by simple random sampling; then, 66 overweight pregnant women were enrolled by convenience sampling and divided into intervention and comparison groups. Intervention group received individual nutritional counselling and physical activity training. The data were collected in several stages with the demographic and obstetric questionnaire, maternal weight record, food frequency and international physical activity questionnaire.

**Results:**

Pregnancy weight gain‐4.75(CI 95%: −4.02, −5.48) was significantly lower in the intervention group (*p* < .001). Comparing between groups with adjustment for baseline values indicated that there was a statistically significant difference in terms of total calorie 95.46 (CI 95%: −22.37, 213.30), carbohydrate 23.45 (CI 95%: 2.12, 44.78), protein −7.16 (CI 95%: −12.85, −1.47) and fat 8.82 (CI 95%: 2.21, 15.67) intake. Despite the higher level of physical activity in the intervention group, there was no statistically significant difference between the two groups.

**Conclusion:**

Counselling interventions for healthy living during pregnancy can lead to controlling weight gain, improving dietary habits and increasing the physical activity in overweight pregnant women.

## 1 INTRODUCTION

Increased prevalence of obesity and overweight in different age groups, especially in women during their reproductive age, is one of the important health problems (Dodd et al., 2014; Jepson, Harris, Platt, & Tannahill, 2010), and disregard for the desired weight gain during pregnancy due to adverse effects on maternal and foetal health is one of the major concerns in prenatal care (Loh, Oen, Koo, Ng, & Yap, 2018). Reports indicate that more than 1.9 billion (39%) of the world's population are considered as overweight and obese, with 40% of them being women (O'Brien, Cramp, & Dodd, 2016). Studies conducted in Iran also show a two‐fold increase in the prevalence of obesity in women (Jafari‐Adli et al., 2014; Kiadaliri, Jafari, Faghihzadeh, Kalantari, & Asadi‐Lari, 2014) in such a way that the prevalence of overweight in women is 27.5%–38.5% and the prevalence of obesity is 12.6%–25% (Jafari‐Adli et al., 2014). According to studies, 50% of women in developed countries have a body mass index above 25 at the time of pregnancy (Dodd et al., 2014; O'Brien et al., 2016). On the other hand, pregnancy is a critical time for women and their children to become overweight and obese (Thangaratinam et al., 2012; Tol, Tavassoli, Shariferad, & Shojaezadeh, 2011; Tovar, Chasan‐Taber, Bermudez, Hyatt, & Must, 2010). Because pregnant women face a myth of "eating for two" and "more rest", are better during pregnancy (Fathnezhad‐Kazemi & Hajian, 2019; Warren, 2013). Therefore, a statistically significant percentage of pregnant women lose control over their eating habits and their emotional eating increases (Kimmel, Ferguson, Zerwas, Bulik, & Meltzer‐Brody, 2016). According to studies, 20 to 40 per cent of women in Europe gain more than recommended weight during the pregnancy (Mourtakos et al., 2015). However, maternal overweight and obesity are associated with increased prevalence of chronic diseases, increased healthcare costs (Gebler, Charuvastra, & Silver, 2015), increased risk of maternal and neonatal mortality, increased risk of gestational diabetes, hypertension in pregnancy, greater use of induction of labour and caesarean section, neonatal macrosomia, hard labour (dystocia), increased probability of admission to intensive care unit and neonatal hyperbilirubinemia (Dodd et al., 2014; Mourtakos et al., 2015). Increased risk of developing type 2 diabetes (Liu, Ao, Yang, & Wang, 2013; O'Brien et al., 2016) and future cardiovascular disease in mother and child are some of the long‐term complications of obesity and high weight gain during the pregnancy (Shah, Retnakaran, & Booth, 2008). Evidence suggests that the complications of excessive weight gain during the pregnancy are directly associated with increased body mass index (BMI) (Dodd et al., 2014). In addition, due to inappropriate diets obese and overweight people face nutritional deficiencies more than people with normal weight, with studies suggesting unbalanced intake of macronutrients and micronutrients in such individuals (Groth & Morrison‐Beedy, 2013; Hui et al., 2012). Proper diet and physical activity are important components of lifestyle; they not only affect pregnancy outcomes but also have intergenerational effects (Lindström, 2012). They also improve maternal and child physical and mental health (A. Hui et al., 2012; Thompson, Vamos, & Daley, 2015). However, interventions based on diet, physical activity and healthy lifestyle had no statistically significant effect on weight loss and improvement of pregnancy outcomes (Phelan et al., 2011). Studies carried out in Iran suggest that pregnant women have poor knowledge about physical activity (Noohi, Nazemzadeh, & Nakhei, 2010) and proper diet during pregnancy (Kamalifard, Charandabi, Mameghani, Jafarabadi, & Omidi, 2012). And since the best interventions to get optimize outcomes for maternal and child are still unknown and research on adopting a healthy lifestyle has not been robust (Oteng‐Ntim, Varma, Croker, Poston, & Doyle, 2012) and the findings are inconsistent (Baheiraei, Mirghafourvand, Mohammadi, & Charandabi, 2012; Groth & Morrison‐Beedy, 2013). Therefore, we performed a cluster randomized trial on a lifestyle intervention for overweight pregnant women.

The research questions were as follows:

1. Is there the efficacy of healthy lifestyle interventions including individual nutritional counselling and physical activity on weight gain during pregnancy in overweight pregnant women?
2. Is there the effectiveness of these interventions on maternal and neonatal outcomes (gestational diabetes, neonatal weight, etc.)?

## 2 METHOD

### 2.1 Study design and

This open‐label, prospective, cluster randomized controlled trial was conducted from 4 April 4–20 January 2018 in Tabriz, Iran. The study had two‐arm parallel‐group design. Tabriz is the fifth largest city of Iran and has 20 health centre in 12 clusters. Randomization was performed at the cluster level. Cluster randomization avoids dissemination bias, which would have occurred if individuals were randomized and treated within the same practice. First, by using Randomizer software, 6 health centres were selected through simple random sampling. Then, three Health centre was randomly selected as intervention centres and three health centre as comparison group. Then at each of them, list of all pregnant mothers meeting the inclusion criteria (first based on Nulliparity then by other criteria) were listed based on demographic data in the records of pregnant mothers and enrolled into study through convenience sampling. Initially, they were called and invited to participate in the study. In case of not participating in the first study session, other eligible participants were requested to join the study to complete the final sample size. For determining the sample size, primary object of the study was used which was the weight gain during pregnancy. The sample size for comparing two groups was calculated by formula below with the confidence interval of 95% and power of 90%. And the effect size was concidred equivalent to similar study (Wolff, Legarth, Vangsgaard, Toubro, & Astrup, 2008), 22 individuals were assigned to each group. Due to three stages of follow‐up and probability of sample loss, sample size increased by 50% and final sample size was 33 in each group and 66 in total. STATA13 software was used to determine sample size:

$$N=\frac{{\left(Z_{1}‐\alpha /2+Z_{1}‐\beta \right)}^{2}{\left(S_{1}^{2}+S_{2}^{2}\right)}^{2}}{\Delta^{2}}$$

Study parameter: alpha = 0.0500, power = 0.9000, delta = −6.7000, m1 = 13.3000,

m2 = 6.6000, sd1 = 7.5000, sd2 = 5.5000, estimated sample sizes’ = 44 so N per group = 22.

### 2.2 Study participants

**Figure 1:**
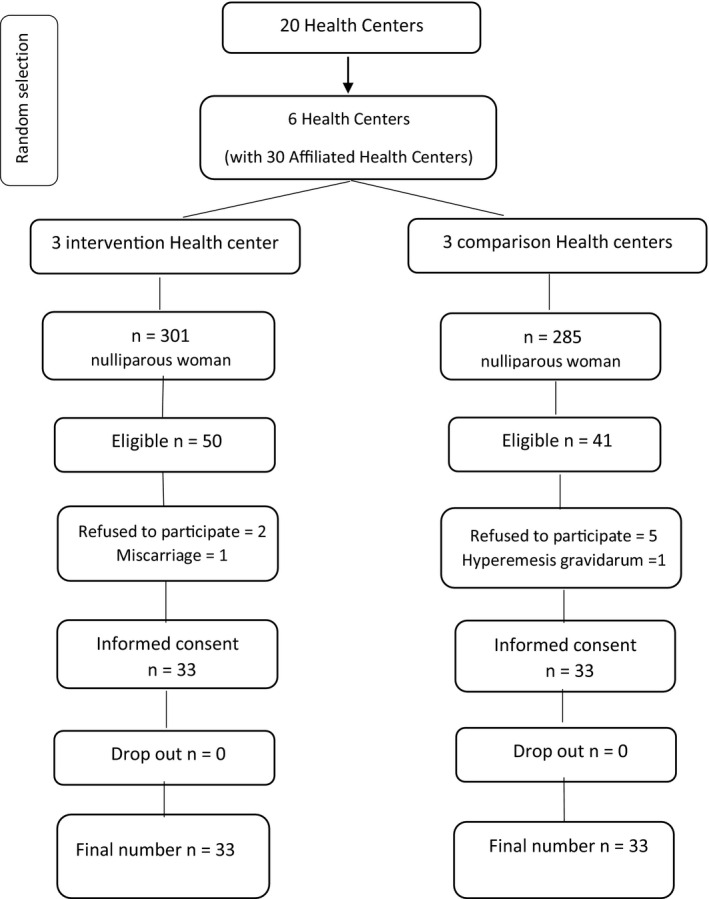
Participant flow

Pregnant women meeting all inclusion criteria including primiparous women with singleton pregnancy with 16–20 weeks of gestational age, BMI 25–29.9, age range 18–40 years and no underlying chronic diseases or risk factors were enrolled into study. Exclusion criteria were taking any type of chemical or herbal drug (other than dietary supplements recommended during pregnancy) that could interfere with normal weight gain during pregnancy, unwillingness of the mother to continue the study or to participate simultaneously. In another studies with similar interventions, emerging of any disorders or gynaecological problems requiring special care for the mother that could interfere with the interventions of the present study and to have an abortion. To reduce the dropout rate of the study, subjects initially were trained and briefed to attend the follow‐up programmes; otherwise, telephone calls were made (Figure 1).

## 3 MEASUREMENTS

### 3.1 Socio‐demographic and pregnancy history

It consists of the demographic variables of pregnant women containing age, educational level, occupational status of pregnant women and their spouses, self‐assessment of household economic status, and midwifery variables, including the first day of the last menstruation, probable due date, gestational age based on first trimester ultrasound, number of pregnancies and childbirths, and height and weight before pregnancy.

### 3.2 Food frequency questionnaire

This method is most efficient dietary assessment method (Ebrahimi‐Mameghani, Behroozi‐Fared‐Mogaddam, & Asghari‐Jafarabadi, 2014). In this study, a 147‐item food frequency questionnaire was used. The questionnaire asked food intake by day, week, month and year over the past year; then, all data were estimated in terms of standard weekly values for each unit or share. The data on each unit of food per day, in grams or millilitres, were entered to Nutrition VI software. The amount of macronutrients energy including carbohydrates, fat, protein and dietary fibre and their portion of total energy intake was estimated and reported.

### 3.3 Physical activity questionnaire

In this study, the short form of the self‐reported International Physical Activity Questionnaire was used. This questionnaire includes 7 questions about the amount of physical activity, and walking (light), the intensity and duration of the activity. Weekly Physical activity longer than 10 min were recorded. According to this questionnaire scoring guide, a person's physical activity level is calculated based on his/ her total physical activity over the past week in MET‐units. In this Questionnaire, light intensity physical activity and walking is considered 3.3 METs, moderate intensity physical activity is 4 METS and vigorous intensity physical activity is 8 METs. To calculate the total physical activity per week, one must sum up the duration of walking (MET × minute × days) with duration of moderate intensity physical activity (MET × minute ×days) and duration of vigorous intense physical activity (MET × minute × days) in a week (Chasan‐Tab er, 2012; Kazemi, Hajian, & Sharifi, 2019).

### 3.4 Intervention protocol and its implementation steps

Most of the interventions in the intervention group included individual counselling as listed below:

1. Prescribing a balanced and flexible diet based on the maternal weight gain pattern during pregnancy, correcting nutritional misconceptions while respecting family cultural beliefs.
2. To help with optimal weight gain during pregnancy, it was recommended to exercise safely through in‐person training, providing pamphlets and educational booklets. Trainings included aerobic exercise at least 3 times a week with moderate intensity and an exercise programme including 5‐min stretching exercises.

A.fnK enrolled clusters and who assigned clusters to interventions, and S.H generated the random allocation sequence. To perform the intervention, after selecting healthcare centres as intervention site, pregnant women meeting the inclusion criteria who were 16–20 weeks pregnant were included in the study and were followed up in three time periods; 26–28 weeks, 35–37 weeks and 7 days postpartum. At the beginning of the study, after randomization and completing written informed consent, participants were provided with demographic, obstetric, food frequency and physical activity questionnaires which were completed in 45–60 min after explanations of the researchers. Then, in the intervention group, the researcher provided the necessary training and counselling on physical activity and nutritional information during pregnancy for the mother for 30 to 45 min depending on the mother's awareness and need. Nutrition and diet consultation was provided by nutritionist to the mothers. The researcher also provided the intervention group with educational booklets and pamphlets that included information on diet and physical activity. Afterwards, mothers were advised to attend follow‐up and weight control sessions during pregnancy. In addition, the researcher shared their contact number with the mothers of the intervention group to respond their self‐care questions any time during the 6‐month intervention period. In each session, counselling was provided depending on the needs of the mothers in the intervention group. To avoid imposing additional costs and problems on commuting to the health centre, attempts were made to co‐ordinate primary interventions and follow‐up visits with prenatal care visits. Therefore, all scheduled visits were concomitant with one of the mother's visits to the health centre. The visits were as follows:

1. First visit (16–20 weeks pregnant): Initially, the weight, height and body mass index of the study units of both groups were measured; also by using their files, baseline weight was recorded. Then, to calculate the energy intake of macronutrients, researcher interviewed the subjects using a food frequency questionnaire, and for evaluating the daily physical activity, physical activity questionnaire (IPAQ) was completed by the research units. VI Nutrition software was used to evaluate the amount of energy and macronutrients (which includes 7 items of energy intake in kcal, carbohydrate, protein and fat per gram and percentage of fat, carbohydrate and protein of total energy intake). After completing the questionnaire, intervention group received guidance on healthy eating and exercise during pregnancy and nutritionist provided individual counselling on daily diet.
2. Second visit (26–28 weeks pregnant): This visit was to track the compliance of the participant with the recommended diets regarding the amount and pattern of gained weight and calculated physical activity level based on IPAQ. During this visit, the participants completed the IPAQ questionnaire for the second time, and the amount of physical activity was calculated. Additionally they were advised to engage in proper physical activities such as aerobic exercise and walking for at least 20–30 min daily at certain intervals of the day (preferably in the afternoon) also in accordance with the provided booklet Stretch trainings were taught to participants. Nutrition counselling was provided if needed. In accordance with the screening instructions and the diagnosis of gestational diabetes, FBS and 2‐hr oral glucose tolerance test (OGTT) were recorded based on mother's files (which were tested in reference centres in each health complex).
3. Third visit (35–37 weeks pregnant): This visit was to track the compliance of the participant with the recommended diets about the amount and pattern of gained weight and calculated physical activity level based on IPAQ

The comparison group was evaluated solely for energy intake and level of physical activity and did not receive any further advice or intervention from the researcher. In accordance to The Integrated Maternal Health Care Guidelines, both groups received routine care during their pregnancy on visits scheduled by their healthcare provider (obstetrician or family health expert) at the health centre. During this study at scheduled dates, the researcher contacted the mothers through telephone calls for tacking their weight and completing the questionnaires. During their last visit, educational pamphlets were given to the mothers of comparison group. Finally, by using a researcher‐made checklist based on the maternal birth file at the neonatal hospital the outcome of childbirth, maternal final weight and maternal and foetal/ neonatal outcome was recorded. Up to 7 days after delivery, additional required information was obtained at the health centre during the first and second postpartum care (3–10 days) visits if not mothers were contacted through phone call. Outcomes were compared between the two groups.

### 3.5 Ethics approval and consent to participate

This study was approved by the Ethics Committee of Shahid Beheshti University of Medical Sciences, Grant no: SBMU.PHNM.1395.498. as part of a PhD dissertation. Written informed consent was obtained from all participants. The study is also registered on the Iranian Registry of clinical Trials website under the registry number IRCT20161122031023N2.

### 3.6 Data analysis

After collecting data from all units, results were analysed using STATA 13 software. To describe the symmetric quantitative data, mean and standard deviation were used. Qualitative data were also reported with frequency and percentage. To compare the qualitative data, chi‐square test was used. Also, considering the design of the study (cluster allocation) generalized linear models with random effects (random‐effects model) were used to compare the mean of macronutrients at two time and physical activity at three‐time points and interaction between time and group was investigated. Pairwise comparisons were performed with the follow‐up test including Tukey. Independent *t* test was also used to compare fasting blood glucose and glucose tolerance test in 24–28 weeks of gestation.

## 4 RESULT

According to the demographic and obstetric characteristics of the studied units, the mean (*SD*) age of mothers was 25.50 (3.84) and most (50%) was in the age group of 25–30 years old. Fifty‐five women (78.2%) had diploma and higher education and 53 women (80.3%) were housewives. Data analysis indicated no statistically significant difference between the demographic characteristics of the two groups, except for height and husband's education (Table 1).

**Table 1:** Demographic and obstetric characteristics of overweight pregnant women in two groups of intervention and comparison

Variable		Intervention group (*N* = 33)	Comparison group (*N* = 33)
		Mean (*SD*)	Mean (*SD*)
Mother age( year)		25.94 (4.22)	25.06 (3.43)
Weight at the start of pregnancy(Kg)		71.69 (5.03)	69.45 (5.05)
Height (cm)		160.72 (4.33)	158.50 (3.93)
BMI		27.73 (1.34)	27.61 (1.19)
Wrist (cm)		16.01 (1.19)	15.96 ( 0.86)
Gestational age at the beginning of the study (week)		18.73 (1.38)	18.42 (1.30)
Variable		*N* (%)	*N* (%)
Maternal educational level	Secondary	14 (53.1)	8 (24.2)
	High school	11 (33.3)	14 (42.4)
	Academic degrees	9 (27.3)	11 (33.3)
Maternal occupational status	Housewife	28 (84.8)	25 (75.8)
	Employed	5 (15.2)	8 (42.2)
Educational level of spouse	Secondary	14 (42.4)	14 (42.4)
	High school	9 (27.3)	15 (45.5)
	University	10 (30.3)	13 (39.4)
Occupational status of spouse	Unemployed	10 (30.3)	7 (21.2)
	Employee	4 ( 12.1)	14 (42.4)
	Self‐employed	19 (57.6)	12 (36.4)

Based on results, mean (*SD*) total calories consumed by pregnant women at the beginning and the end of the study were 2,100 (236) and 2,337 (238), respectively. Comparison of mean calorie, carbohydrate and protein intake before the intervention in the two groups showed no statistically significant difference; however, the intervention group had higher fat intake and the difference between two groups was statistically significant (*p* = .009). In spite of the fact that the amount of calories consumed at the end of the study was lower in the intervention group, there was no statistically significant difference between two groups (*p* = .112) but after adjustment baseline values comparison between two groups was statistically significant (*p* < .001). Also, there was a statistically significant difference in carbohydrate, protein and fat intake, indicating an increase in protein and a decrease in fat intake in the intervention group. Also, the amount of dietary fibre intake increased in the intervention group compared with the beginning of the study which was significantly (*p* < .001) higher than control group. Data analysis indicated that there was a statistically significant difference between the two groups in terms of dietary protein intake at the end of the study (*p* < .001). This difference was not significant at the start of the study indicating an increase in protein intake in the intervention group. However, the ratio of carbohydrate to total consumed calories at the end of the study was not statistically significant (*p* = .617). However, consumption in the intervention group was lower and had more fibre. Also, the intervention group had more calcium intake than the control group (*p* < .001) (Table 2). Although the level of activity was higher in the intervention group, especially during the study (second trimester of pregnancy), there was no statistically significant difference (*p* = .7) between the two groups at three different times (Table 3).

**Table 2:** Mean and *SD* daily consumption of food in pregnant women at the beginning and end of the study

Receive daily	Intervention group (*N* = 33)^a^		Comparison group (*N* = 33)^a^		95%CI (MD)		*p* values		*p* values^b^
	Beginning of the study (16–20 week)	End of the study (35–37 week)	Before intervention (16–20 week)	After intervention (35−37week)	A	B	A	B	
Total calorie	2074.79 (224.65)	2,384.97 (239.19)	2,126.91 (248.27)	2,290.96 (232.50)	−51.29 (−163.78, 61.20)	95.46 (−22.37, 213.30)	.372	.112	<.001
Carbohydrate (g)	291.97 (48.26)	329.06 (44.26)	276.91 (50.44)	305.61 (45.49)	15/06 (−8.39, ‐ 38.51)	23.45 (2.12,44.78)	.208	.031	.044
Protein (g)	74.42 (13.38)	76.39 (13.75)	70.53 (13.31)	83.55 (9.89)	3.89 (−2.44,10.24)	−7.16 (−12.85, −1.47)	.229	.014	<.001
Fat (g)	74.43 (12.86)	91.03 (15.26)	84.95 (18.39)	82.20 (12.43)	−10.52 ( −18.06,−2.71)	8.82 (2.21,15.67)	.006	.009	<.001
Saturated fat (g)	24.51 (8.29)	25.49 (6.31)	26.99 (5.78)	24.21 (4.69)	−2.48 (−5.88, 0.91)	1.28 (−1.36, 3.92)	.152	.342	.181
Cholesterol (g)	192.98 (52.86)	226.22 (69.89)	221.37 (48.68)	194.10 (43.96)	−28.39 (−52.53, −4.24)	32.11 (4.37, 59.85)	.021	.023	.010
Fibre	43.84 (9.73)	46.20 (8.13)	48.46 (9.10)	57.82 (7.43)	−4.62 (‐ 9.10, −0.14)	−11.62 (−15.59, −7.79)	.043	<.001	<.001
Carbohydrate ratio (%)	56.22 (6.94)	55.21 (5.37)	51.98 (5.97)	53.25 (4.58)	4.24 (1.16, 7.32)	1.96 (−0.40, 4.33)	.007	.105	.617
Protein ratio (%)	14.47 (2.82)	12.88 (2.34)	13.38 (2.66)	14.67 (1.85)	1.08 (−0.22, 2.38)	−1.79 (−2.79, −0.79)	.104	<.001	<.001
Fat ratio (%)	32.38 (5.37)	34.42 (5.20)	35.96 (6.45)	34.29 (3.45)	−3.58 (−6.40, −0.76)	2.12 (0.22, 4.22)	.013	.048	<.001
Calcium (mg)	934.75 (304.63)	859 (282.76)	1,076.62 (311.76)	1,192.16 (285.48)	−141.87 (−288.32, 4.57)	−332.22 (−467.22,−197.22)	.058	<.001	<.001

Note

A: before intervention; B: after intervention.

^a^ Mean(*SD*).

^b^ To compare between groups with adjustment for baseline values.

**Table 3:** Mean and standard deviation of physical activity of pregnant women in intervention and comparison groups

Variable	Research steps	Intervention group^b^ (*N* = 33)	Comparison group^b^ (*N* = 33)	95%CI (MD)	*p* values ^a^	Time
Physical activity (MET)	(16–20 week)	1,163 (517)	1,223 (440)	−60.50 (−296.85.175.85)	.7	0.07
	(26–28 week)	1,215 (384)	1,151 (323)	64.57 (−110.09, 239.24)		
	(35–37 week)	1,130 (291)	1,023 (328)	106.78 (−46.07, 259.64)		

^a^ Generalized linear model with random effect.

^b^ Mean(*SD*).

Based on the data related to weight gain in both groups, overall mean weight gain in the intervention group was statistically significant compared with the control group and the weight gain showed less growth (*p* < .001). Also differences in weight gain averages were statistically significant in the second and third trimesters (except first trimester); in spite of the fact that the weight gain of 5 women in the intervention group was in recommended range of IOM, there was no statistically significant difference between the two groups (*p* = .057) (Table 4).

**Table 4:** Comparison of gestational weight gain in the intervention and comparison groups

Variables		Intervention group^a^ (*N* = 33)	Comparison group^a^ (*N* = 33)	95%Cl (MD)	*p* value^c^
Weight gain^a^ (Kg)	Total	12.92 (1.27)	17.67 (1.66)	−4.75 (−5.48, −4.02)	0.001
	First trimester	1.83 (0.81)	2.20 (0.99)	−0.36 (−0.81, 0.07)	.104
	Second trimester	4.97 (0.71)	7.09 (0.82)	−2.12 (−2.50, −1.74)	>.001
	Third trimester	6.11 (0.75)	8.37 (0.78)	−2.26 (−2.64, −1.88)	>.001
Weight gain according (IOM)^b^	At the recommended level	5 (15.2)	0	*p* values^d^	
	More than recommended	28 (84.8)	33 (100)	.057	

^a^ Mean(*SD*).

^b^ *N* (%).

^c^ Generalized linear model with random effect.

^d^ Fisher's exact test.

Also, according to the results of Table 5, there was no statistically significant difference between the two groups in terms of mean fasting blood glucose and glucose tolerance test. Fisher test showed no statistically significant difference between two groups in terms of gestational diabetes (*p* = .56) (Table 5).

**Table 5:** Comparison of mean FBS, GTT1, 2 and frequency of gestational diabetes in two groups of intervention and comparison

Variable		Comparison group (*N* = 33)	Intervention group (*N* = 33)	MD (CI 95%)	*p* values^a^
		Mean (*SD*)	Mean (*SD*)		
FBS at the beginning of pregnancy		81.45 (4.2)	80.78 (6.1)	−0.69 (3.30, 1.90)	.595
(24–28 week)	FBS	85.24 (5.1)	80.88 (4.72)	−1.36 (−3.79, 1.07)	.267
	GCT_1_	165.18 (9.3)	163.52 (5.6)	−1.66 (−5.47, 2.13)	.385
	GCT_2_	131.61 (10.3)	127.76 (8.8)	−3.84 (−8.57, 0.88)	.109
Pregnant women with Gestational diabetes		*N* (%)	*N* (%)	*p* value^b^	
		2 (6.1)	1 (3)	.562	

^a^ Independent sample *t* test.

^b^ Fisher's exact test.

Based on the independent samples *t* test, there was no statistically significant difference between birth weight, height and head circumference of neonates. In addition, caesarean delivery was the same in both groups and the most common cause was elective or planned caesarean section, although this variable was higher in the control group but there was no statistically significant difference in two groups (*p* = .81). Analysis of data indicated that there was no statistically significant difference in neonatal jaundice up to 7 days after delivery (*p* = .32). Other outcomes such as preterm labour, uterine atony and severe postpartum haemorrhage were not observed in any of the groups (Table 6).

**Table 6:** Pregnancy and neonatal outcomes in two intervention and comparison groups

Variable	Comparison group (*N* = 33)	Intervention group (*N* = 33)	MD (CI 95%)	*p* values^a^
	Mean (*SD*)	Mean (*SD*)		
Neonatal weight	3.41 (0.24)	3.30 (0.20)	−0.10 (−0.21, 0.004)	.060
Neonatal height	51.45 (2.43)	51.87 (2.93)	0.42 (−0.90, 1.75)	.525
Head circumference	33.12 (0.67)	33.18 (0.80)	0.06 (−0.30, 0.42)	.742
Variable	*N*(%)	*N* (%)	*p* values^b^	
Caesarean	10 (30.3)	10 (30.3)	1	
Cause of caesarean	Elective	8 (80)	7 (70)	.816
	Bradycardia	1 (10)	1 (10)	
	Progress failure	2 (20)	1 (10)	
Jaundice	17 (5.5)	12 (36.4)	0.321	

^a^ Independent sample *t* test.

^b^ Chi‐square.

## 5 DISCUSSION

Our intervention programme which was an individual counselling based on proper diet and physical activity during pregnancy in overweight pregnant women leads to lower weight gain comparing to our comparison group. However, various studies have inconsistent results about efficacy of lifestyle interventions on weight gain in obese and overweight women (Althuizen, Van Der Wijden, Van Mechelen, Seidell, & Van Poppel, 2013; Hui et al., 2014; Olson, 2007). Polly and et al reported that education on weight gain and physical activity during pregnancy reduces weight gain only in women with normal weight and has no effect on obese and overweight women. According to these researchers, women with high body mass index tend to be more resistant to these interventions (Polley, Wing, & Sims, 2002), despite of the fact that some studies have suggested the positive effects of education and motivational programmes on weight control in obese women. In a case–control randomized experimental study performed in Sweden, in obese pregnant women with additional visits, motivational programmes had a positive effect of on encouraging regular aerobic exercise, developing motivational habits and behaviours and balancing the energy intake and appropriate weight gain during pregnancy (Claesson et al., 2008). A quasi‐experimental study was conducted by Mottola and et al for modifying the health promoting behaviours in obese and overweight pregnant women; energy intake was limited to 2000 calories per a day, the frequency of walking was increased to 3 to 4 times a week. Findings indicated the effectiveness of regular physical activity programme in controlling weight gain during (Mottola et al., 2010) pregnancy in overweight or obese women. In this study, investigation in the influence of education in overweight pregnant women on controlling macronutrient intake indicated, regardless of the fact that at the beginning of the study, before the intervention, total calories, carbohydrate and protein levels did not differ between the two groups there was a statistically significant difference in fat intake and the intervention group had higher fat intake. The results after the intervention indicated that despite the decrease in calorie intake in the intervention group at the end of the study, there was no statistically significant difference between two groups. Nonetheless, a review of food consumption in the intervention group, indicated a significant increase in protein, fiber and calcium intake and a significant decrease in fat intake. The lack of a significant difference in calories intake might be due to IOM recommendations which does not differentiate between daily calorie intake in normal weight, overweight and obese women, and the recommended amount is 300 calories a day (Moore Simas et al., 2013), and in the present study, this amount was considered in dietary recommendations, and the goal was to promote healthy eating culture and to control optimal weight gain. Additionally, in given consultation it was advised to use reduce consumption of saturated fats and carbohydrates and to increase the unsaturated fats, protein intake. Also, it was recommended to use fruits, vegetables and fibres more commonly so that most of the calories would be provided by these nutrients. Hui et al. conducted a study for investigating the effectiveness of healthy lifestyle interventions, including home exercise, walking 3 to 5 times per week and nutritional counselling in primiparous and non‐diabetic women. They concluded that calorie, triglycerides, cholesterol and saturated intake only decrease in women with normal weight (Hui et al., 2014).

Only in two studies conducted by Wolf and Mottella, the calorie amount was decreased which was due to limited calorie intake (Mottola et al., 2010; Wolff et al., 2008), despite of the fact that the gained weight in our study was lower in intervention group; only 5 pregnant women have reached the recommended weight for overweight women by IOM (7–11.5 kg) and the rest has gained more than the recommended range. Similar diet programmes for total calorie intake in obese, overweight and normal weighted women might be a cause for improper weight gain. Because IOM has the same advise about calorie intake in obese and overweight women (Mottola et al., 2010). It seems that following a treatment and having a regular physical activity might affect the weight gain pattern.

According to the results of this study, despite of the statistically significant increase of physical activity in intervention group in second trimester, there was no change in third trimester. In general, studies conducted for evaluating the effectiveness of counselling and educating the pregnant women to encourage exercising and physical activity had inconsistent results (Althuizen et al., 2013; Hui et al., 2014; Wolff et al., 2008). In the study conducted by Tomoda et al investigating the effect of educational intervention on physical activity during the pregnancy, however, there was no significant change in physical activity (moderate to vigorous level) in the intervention group (Tomoda, Ogita, & Tamura, 1996). Unlike Tomoda, Hui et al. found that physical activity increased significantly two months after the intervention (Hui et al., 2014). Also, Shakeri and colleagues found a statistically significant difference between the two groups by forming 8 training sessions (Shakeri, Fekri, Shahnavaz, & Shakibazadeh, 2012). Chasan‐Taber and et al concluded in their study that physical exercises during pregnancy can significantly increase women's physical activity scores (Chasan‐Tab er, 2012). A study conducted by Heffernan on the effect of relaxation training in primiparous women showed that after education of relaxation training and safe exercise during pregnancy, there was statistically significant difference in mean score of physical activity before and after training (Heffernan, 2000). In a similar study by Mirmulaei and his colleagues, the mean scores of physical activity before training in the two groups showed no statistically significant difference, but after training, the difference was statistically significant (Mirmolaei, Moshrefi, Kazemnejad, Farivar, & Morteza, 2009). It seems that the type of educated activity, the right time to encourage and decision to change behaviour are also effective in the intervention results, so that specific training for each pregnancy trimester that is easy, more accessible and at the same time more acceptable could be more effective than general recommended activities. It seems that the type of training activity and the right time to motivate and decide to change behaviour also influence the outcome of the intervention so that training for any pregnancy course that is easier, more accessible and at the same time more acceptable. It can be more effective than recommended public activities especially in late pregnancy, the time when the mother has less acceptance for such activities. Regardless of the fact, there was no statistically significant difference in pregnancy outcomes between two groups. The study began with the assumption that by designing heath promoting interventions based on supply and demand and making them safe and more accessible at the same time, it would prevent some of the probable complications of overweight and obesity (Campbell, Johnson, Messina, Guillaume, & Goyder, 2011).

According to systematic reviews and meta‐analysis of studies related to healthy lifestyle‐promoting interventions, these interventions were associated with appropriate weight gain pattern of mothers in pregnancy; however, there was no statistically significant difference in pregnancy outcomes between the study groups.

### 5.1 Limitation

One limitation of the study was assuming that pregnant women will not be able to follow regular dietary or exercise regimes in the first trimester due to some common complaints such as nausea, vomiting, feeling tired and requiring more sleep. Participants were selected from mothers with 16–20 weeks of gestational age. However, it is recommended to begin interventions earlier and if possible before the pregnancy.

## 6 CONCLUSION

Pregnancy is an opportunity to identify healthy behaviours and to reinforce or motivate them to change health‐related misconducts. However, the results of the third sub‐study suggest that despite some differences observed between women who received counselling and training to improve nutrition and physical activity with those who did not receive such trainings, the difference in optimal weight gain, based on world‐class recommendations, occurred in a handful of pregnant women. However, statistically significant differences in the pattern of healthy eating and preventing overweight in the intervention group were promising, and it improves the efficacy of these interventions.

## CONFLICT OF INTEREST

The authors declare that they have no competing interests.

## AUTHORS’ CONTRIBUTION

A.fnK contributed to developed of the concept, collected data, analysed data and wrote the draft and final article. S.H contributed to development of concept and reviewed the draft and final article. A.A contributed to collected data and wrote the draft and final article. P.S contributed to analysed data.
